# Phosphorus in Peritonitis—A Verification

**Published:** 1898-02

**Authors:** Howard Crutcher

**Affiliations:** Chicago


### PHOSPHORUS IN PERITONITIS—A VERIFICATION.

Howard Crutcher, M. D., Chicago.

The day after Christmas, 1897, Loretta M., a vivacious Irish girl of sixteen, was taken with severe abdominal cramps while riding in a street car. After arriving at home, a few moments later, she vomited twice. The next day a sharp, prolonged chill came on, and the case came under the care of the Dunham College Dispensary. The Dispensary physician sent for me the day the chill came on. While a prompt diagnosis of perforating appendicitis was given, the patient's family were so violently opposed to surgery that I advised nothing of an operative character. My judgment, expressed to the attendant, was that a fatal termination from sheer neglect was about all that could be expected.

Three days later the condition of the girl was so critical that her family clamored for an operation. If I had possessed a shadow of regard for my "per cent." record I would not have gone near the case with a satchel of instruments. A firm "tumor" was located in the right iliac region. The mechanical difficulties of the operation were very trying. The gut was glued firmly to the abdominal wall anteriorly, and the abscess was located behind the cæcum. A heroic slash with a beautiful bistoury would have laid the gut wide open for a couple of inches, but no pus would thereby have been found. The contents of the abscess nowhere appeared against the anterior parietal peritoneum. The adhesions were so frail that the general cavity got a wholesale dose of pus. Prolonged flushing brought out about half a pint of fetid pus and a great deal of flaky lymph. After the insertion of a large Mikulicz drain the patient was put to bed. The shock was not threatening.

Up to the fourth night the progress of the case was far more favorable than I had dared to hope it would be. About the beginning of the fifth day the crash came with a vengeance. The pulse was 130, the mind was wandering, the urine and feces passed without restraint, and it was very evident that the girl was rapidly sinking. *Arsenic* was given repeatedly, but without avail. I sent a message to the students who were nursing the case, to the effect that death was inevitable, but that a hot saline enema might prolong life.

On attempting to give the enema the rectum was found to be open, and no resistance whatever was offered by the sphincter ani. Grayish-white fecal discharges, watery and offensive, passed constantly. The students recognized the indications for *Phosphorus,* gave a dose of that remedy, and, instead of dying, the girl got well.

This was the most remarkable recovery that I have ever seen in septic peritonitis. I am not foolish enough to believe that many such cases ever will recover, but this case did, and the diagnosis was so absolute, the prognosis so desperate, and the result obtained so conclusive that the skeptic who doubts it would not believe though one arose from the dead. The improvement was not of that instantaneous character so popular in imaginative literature, but it was clear-cut and amply convincing.

I have nearly fallen out of the habit of reporting my recoveries in appendicitis operations, and this case would not be given were it not for the convincing evidence it affords of the priceless value of rational therapeutics in desperate surgical cases.

